# Atomically resolved Au_52_Cu_72_(SR)_55_ nanoalloy reveals Marks decahedron truncation and Penrose tiling surface

**DOI:** 10.1038/s41467-020-14400-2

**Published:** 2020-01-24

**Authors:** Yongbo Song, Yingwei Li, Hao Li, Feng Ke, Ji Xiang, Chuanjun Zhou, Peng Li, Manzhou Zhu, Rongchao Jin

**Affiliations:** 1 Department of Chemistry and Centre for Atomic Engineering of Advanced Materials, Anhui Province Key Laboratory of Chemistry for Inorganic/Organic Hybrid Functionalized Materials, Anhui University, 230601 Hefei, Anhui People’s Republic of China; 2 Key Laboratory of Structure and Functional Regulation of Hybrid Materials, Anhui University, Ministry of Education, 230601 Hefei, Anhui People’s Republic of China; 3 Department of Chemistry, Carnegie Mellon University, Pittsburgh, PA 15213 United States

**Keywords:** Nanoparticles

## Abstract

Gold-copper alloys have rich forms. Here we report an atomically resolved [Au_52_Cu_72_(*p*-MBT)_55_]^+^Cl^−^ nanoalloy (*p*-MBT = SPh-*p*-CH_3_). This nanoalloy exhibits unusual structural patterns. First, two Cu atoms are located in the inner 7-atom decahedral kernel (M_7_, M = Au/Cu). The M_7_ kernel is then enclosed by a second shell of homogold (Au_47_), giving rise to a two-shelled M_54_ (i.e. Au_52_Cu_2_) full decahedron. A comparison of the non-truncated M_54_ decahedron with the truncated homogold Au_49_ kernel in similar-sized gold nanoparticles provides for the first time an explanation for Marks decahedron truncation. Second, a Cu_70_(SR)_55_ exterior cage resembling a 3D Penrose tiling protects the M_54_ decahedral kernel. Compared to the discrete staple motifs in gold:thiolate nanoparticles, the Cu-thiolate surface of Au_52_Cu_72_ forms an extended cage. The Cu-SR Penrose tiling retains the M_54_ kernel’s high symmetry (*D*_5h_). Third, interparticle interactions in the assembly are closely related to the symmetry of the particle, and a “quadruple-gear-like” interlocking pattern is observed.

## Abstract (web summary)

The formation of Marks truncated decahedra in nanoparticles is ubiquitous but the mechanism has not been fully understood. Here, the authors provide atomic-level insights by creating a non-truncated Au_52_Cu_72_(SR)_55_ decahedral nanocluster and comparing it with the truncated homogold decahedra.

## Introduction

Multiply-twinned nanoparticles (MTPs) are particularly important and intriguing in nanoscience research^1–3^, but the formation mechanism has long remained unclear. Historically, Ino and Ogawa first found in the 1960 s MTPs in the early stages of gold film growth^4^, and such particles could be viewed as 5 or 20 face-centered-cubic (fcc) tetrahedra joined together with twin boundaries, forming decahedra or icosahedra. Marks and Howie reported the presence of such MTPs up to 200 nm in various heterogeneous metal particles (silver in particular)^5^. In their further work, thermally annealed Au or Ag nanoparticles of 10–50 nm showed re-entrant surfaces on the decahedral MTPs^6^, and the truncated-decahedron was proposed^7,8^, which costs less energy than the truncation in the Ino decahedron.

With the advent of the nanotechnology era, metal nanoparticles (NPs) have since drawn significant attention, and capabilities for manipulating the structure and properties of NPs have been achieved to a great extent^1–3,9,10^. Monodispersed decahedral NPs (e.g., 50–100 nm) can be produced with excellent control^11,12^. With respect to small NPs (e.g., gold) in the 1–3 nm regime, the Marks truncated-decahedron is preferentially formed^8^, as it is energetically favored based on atomistic modeling^13^, but no atomic-level experimental insight has been obtained yet.

Recent progress in controlling NPs with atomic precision has opened new exciting opportunities^14^, as the total structure (kernel + ligands) of such NPs can now be solved by single crystal X-ray diffraction (SC-XRD). The total structure reveals not only the atomically resolved core structure, but also the surface organic ligands and the organic-inorganic interface^14^. Research progress has led to a number of small-sized NPs with solved structures, but the structural determination of larger-sized ones (>100 metal atoms) still remains challenging, yet their structural information is critical for understanding the fundamental science ranging from bonding to symmetry, kernel packing to staple-motif protection, surface energy and facet exposure, as well as optical applications^14,15^. When comparing the homogold NPs of more than 100 atoms, one can find that truncation is ubiquitous in their kernel structures, such as the Marks-decahedra in Au_102_(SR)_44_ and Au_103_S_2_(SR)_41_^16,17^, the Ino decahedra in Au_130_(SR)_50_ and Au_246_(SR)_80_^18,19^. However, it still remains unclear to date whether a full or perfect decahedral kernel can be realized, and how the truncation occurs, albeit some factors such as the surface stress effects and elastic strain energy effects have been invoked and discussed^20–22^.

High-symmetry kernels are ubiquitously observed in thiolate-protected gold NPs, such as *D*_5h_ kernels, and their surfaces are generally protected by discrete staple motifs such as monomers and dimers^14^. The surface pattern formation of motifs reduces the kernel’s *D*_5h_ to lower symmetry for the entire particle, which often endows chiral symmetries such as *D*_5_^18,19^, and *C*_*n*_^16,17,23^. The symmetry-breaking by the arrangement of surface motifs is to relieve the steric crowding of the ligands in large sizes^24^. Relaxation is made possible by the low-coordinated sulfur in staple motifs, usually a coordination number *μ* = 2 (i.e., with two S–Au bonds), making them discretely arranged on the surface of Au NPs. Such a surface pattern is universally adopted in all cases of solved gold structures^25,26^. However, it remains unclear in the case of nanoalloy systems. Therefore, we are motivated to pursue the Au-based alloy system for novel structural characteristics arising from heterometal incorporation and for deeper understanding of the growth pattern, truncation, and other major issues in metal NPs. The Au–Cu is chosen, for that its bulk phase behavior has been well understood^27^, but the nanoscale system remains largely under-explored^28^.

Herein, we report a well-defined [Au_52_Cu_72_(*p*-MBT)_55_]^+^ (*p*-MBT = SPh-*p*-CH_3_) nanoalloy with unusual distribution of Cu atoms and the resultant consequences. Instead of forming a usual distribution of dopants, we find that Cu atoms go to specific sites: two Cu atoms are found in the innermost decahedral M_7_ kernel and 70 copper atoms are on the surface. The incorporation of Cu atoms into the innermost part of the nanoalloy is against the well-known rule that atoms of lower electronegativity should appear on the surface. Significantly, the two specific Cu atoms lead to a shortening of the *C*_5_ axial Au–Cu–Cu–Au distance by 7% compared to the Au–Au–Au–Au axis observed in the truncated decahedral Au_49_ kernel of homogold NPs. The relaxed strain in the Cu_2_Au_52_ kernel is identified to be critical for the formation of the full decahedral Au_52_Cu_2_ kernel, as opposed to the formation of a Marks-decahedron. The entire nanoalloy shows an unprecedented high symmetry (*D*_5h_) from the kernel to the surface, with the surface pattern resembling an aesthetic three dimensional (3D) Penrose tiling. Such a high symmetry is ascribed to the outer Cu-thiolate cage—which is rigid due to all highly coordinated sulfur. The interparticle interactions in the assembly of this nanoalloy exhibit a “quadruple-gear” interlocking pattern via tip-to-edge and edge-to-edge interactions between the ligands of nearest pentagonal particles. The revealed truncation for Marks-decahedron, *D*_5h_ symmetry retention from kernel to surface, and the assembly pattern of [Au_52_Cu_72_(*p*-MBT)_55_]^+^ may open new opportunities in future nanoalloy research.

## Results

### Synthesis and characterization

The [Au_52_Cu_72_(*p*-MBT)_55_]^+^ nanoalloy was synthesized by co-reduction of a mixture of Au(I) and Cu(II) precursors by NaBH_4_. Briefly, thiol was first added to a mixture of AuPPh_3_Cl and CuCl_2_ in a toluene/ethanol solution, followed by NaBH_4_ reduction. After washing with CH_3_CN and extraction with dichloromethane, a pure product was obtained (see Method for details). Black, rod-like crystals were obtained by diffusing CH_3_CN into a toluene solution for 6−10 days at 10 °C, and then characterized by X-ray crystallography. The product was identified to be [Au_52_Cu_72_(*p*-MBT)_55_]^+^ with Cl^−^ as its counterion, and the macroscopic single crystal is in space group *P*$\bar 1$ (Supplementary Table 1).

**Fig. 1:**
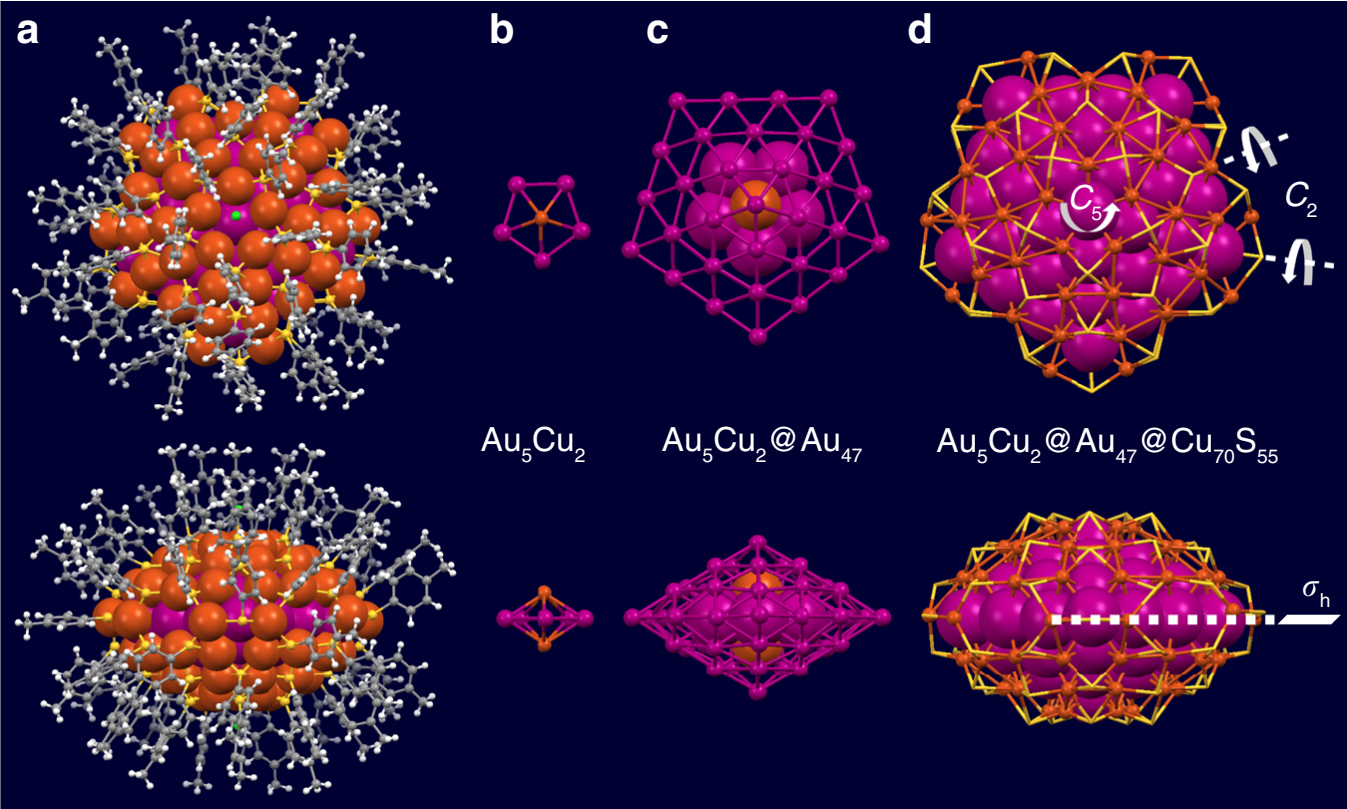
Structural anatomy of the [Cu_72_Au_52_(*p*-MBT)_55_]^+^ nanoalloy. **a** total structure of [Cu_72_Au_52_(*p*-MBT)_55_]^+^ in top/side views; **b** the first Au_5_Cu_2_ shell; **c** the second Au_47_ shell; and **d** the third Cu_70_S_55_ cage. Color labels: magenta = Au, orange = Cu, yellow = S, grey = C, and white = H.

Figure 1 shows the dissection of the [Au_52_Cu_72_(SR)_55_]^+^ nanoalloy, which exhibits a pentagonal shape (Fig. 1a) and comprises three shells: the first Au_5_Cu_2_ shell of *D*_5h_ symmetry (Fig. 1b), the second Au_47_ shell (Fig. 1c), and the third shell—a Cu-thiolate cage that resembles a five-petal flower (Fig. 1d). Some distortions are found in the cage, but one can still consider that there is one quasi-*C*_5_ axis, ten quasi-*C*_2_ axis perpendicular to the quasi-*C*_5_ axis, and one horizontal symmetry-plane in the structure, hence, an overall quasi-*D*_5h_ symmetry.

Additional analyses were performed to confirm the metal composition of this nanoalloy. The composition of [Cu_72_Au_52_(*p*-MBT)_55_]^+^ was verified by inductively coupled plasma mass spectrometry analysis (ICP-MS). A molar ratio of Cu:Au (72:52) was found in all three measurements (Supplementary Table 2), which is consistent with the X-ray crystallographic result.

Mass spectrometry (MS) via matrix-assisted laser desorption ionization (MALDI) and laser desorption ionization (LDI) (Supplementary Figs. 1–3) was further employed to analyze the alloy nanoclusters. It should be noted that the nanocluster formula was determined by X-ray crystallography, while MALDI and LDI are not accurate for formula determination of large nanoclusters (>100 metal atoms) due to the often occurred fragmentation (e.g., loss of surface ligands). Nevertheless, intensity-dependent MALDI/LDI mass spectra for two samples (protected by *p*-MBT and TBBT (where, TBBT = 4-*tert*-butylbenzenethiolate), respectively) show peaks at similar *m/z* (close to the MW of M_124_S_55_) (Supplementary Figs. 2, 3, Table 3), which are consistent with the X-ray crystallography analysis.

With cesium acetate added to the nanoalloy solution, adducts with cesium ions (Cs^+^) were formed in the electrospray process and, thus, mass peaks of the nanoalloy were observed. We found that the mass spectrum of TBBT-protected [Au_52_Cu_72_(TBBT)_55_]^+^ shows a better resolution than that of *p*-MBT-protected [Au_52_Cu_72_(*p*-MBT)_55_]^+^ (Supplementary Fig. 4). According to the one-copper-atom difference between two adjacent peaks (i.e., 32 Da (charge *z* = 2), 21 Da (*z* = 3), and 16 Da (*z* = 4)) (Supplementary Fig. 4 B–D), the observed three sets of peaks are assigned to [M + Cs]^2+^, [M + 2Cs]^3+^, and [M + 3Cs]^4+^. A list of peak masses is provided in Supplementary Table 4. Of note, a similar phenomenon (i.e., adjacent sizes with one Au difference) was previously observed in Au_103_(SR)_45_ and Au_104_(SR)_45_^29^.

The nanoalloy surface being all Cu (as opposed to mixed Cu and Au) is also confirmed. First of all, as shown in the zoom-in peaks of Au_52_Cu_72_ (Supplementary Fig. 4 B–D), the difference between adjacent peaks discussed above corresponds to the *m/z* for Cu atom (63.5 Da) as opposed to the difference between Cu and Au atoms (197 − 63.5 = 133.5), indicating the successive detachment of surface Cu atoms without any proportion of Au/Cu. Second, X-ray crystallography can easily tell between gold and copper atoms according to their largely different electron densities. Third, the cage formed by surface Cu and S atoms is in contrast to the discrete staple motifs for the case of Au–SR^25,26^. The detachment of surface copper without losing sulfur in ESI-MS analysis can be explained by the high coordination number of sulfur (Supplementary Fig. 5): upon losing Cu atom(s) (green circles), the S atoms (red circles) are still coordinated by other surface metal atoms.

The Cl^−^ ion observed in the crystal structure analysis is determined to be the counterion of [Au_52_Cu_72_(*p*-MBT)_55_]^+^, rather than a coordinating ligand^30–32^. To further prove this, we used [SbF_6_]^−^ to replace the Cl^−^, followed by re-crystallization of the [Au_52_Cu_72_(*p*-MBT)_55_][SbF_6_], but the quality of the single crystals is not high enough to be resolved. MALDI-MS analysis shows the same peaks in the high mass range (Supplementary Fig. 6A), confirming that the sample is stable after adding NaSbF_6_, and the low-mass range shows peaks at 234.65 and 236.65 Da corresponding to [SbF_6_]^−^ for the sample of [Au_52_Cu_72_(*p*-MBT)_55_][SbF_6_] (Supplementary Fig. 6B), demonstrating that this nanoalloy possesses a + 1 charge state. Thermogravimetric analysis shows a weight loss of 32.12% (Supplementary Fig. 7A), in good agreement with the theoretical value of [Cu_72_Au_52_(*p*-MBT)_55_]^+^ (31.38%). All these results are consistent with the results of X-ray structural analysis.

The UV-vis absorption spectrum of [Cu_72_Au_52_(*p*-MBT)_55_]^+^ (Supplementary Fig. 7B) exhibits five step-wise peaks at 365, 396, 470, 578, and 720 nm, indicating that this nanoalloy is not plasmonic, which is due to quantum confinement caused by its small size. It is also worth noting that this nanoalloy possesses 68 free valence electrons (i.e., 124 – 55 – 1 = 68), which is a closed-shell electronic structure^33^. For the potential ligand effect, we found no discernable difference in the spectra of the two [Cu_72_Au_52_(SR)_55_]^+^ protected by different ligands (Supplementary Fig. 8). The Au_52_Cu_72_ nanoalloy also exhibits good stability (Supplementary Fig. 9).

### Insights into the Marks decahedron truncation

Cu and Au are highly mixable, albeit their atomic radii differ by ~12% (Cu: 1.28 and Au: 1.44 Å). The segregation of two Cu atoms into the M_7_ kernel of the Cu_72_Au_52_ nanoalloy is unusual in ligands-capped metal NPs. In the absence of ligands, Au would segregate to the surface^34^, as opposed to the observed Cu surface in the ligand-protected case. This is because atoms of lower electronegativity usually go to the surface like the outmost Cu-thiolate cage in this case, and the literature work also reported such a trend^35^, albeit theoretical work indicated a possibility of Cu location in the center^36^. The peculiar arrangement of copper atoms at central sites is found to be decisive to form the second, perfect decahedral shell of gold as opposed to a truncated-decahedron for homogold NPs such as Au_102_, Au_103_, and Au_130_^16–18^.

**Fig. 2:**
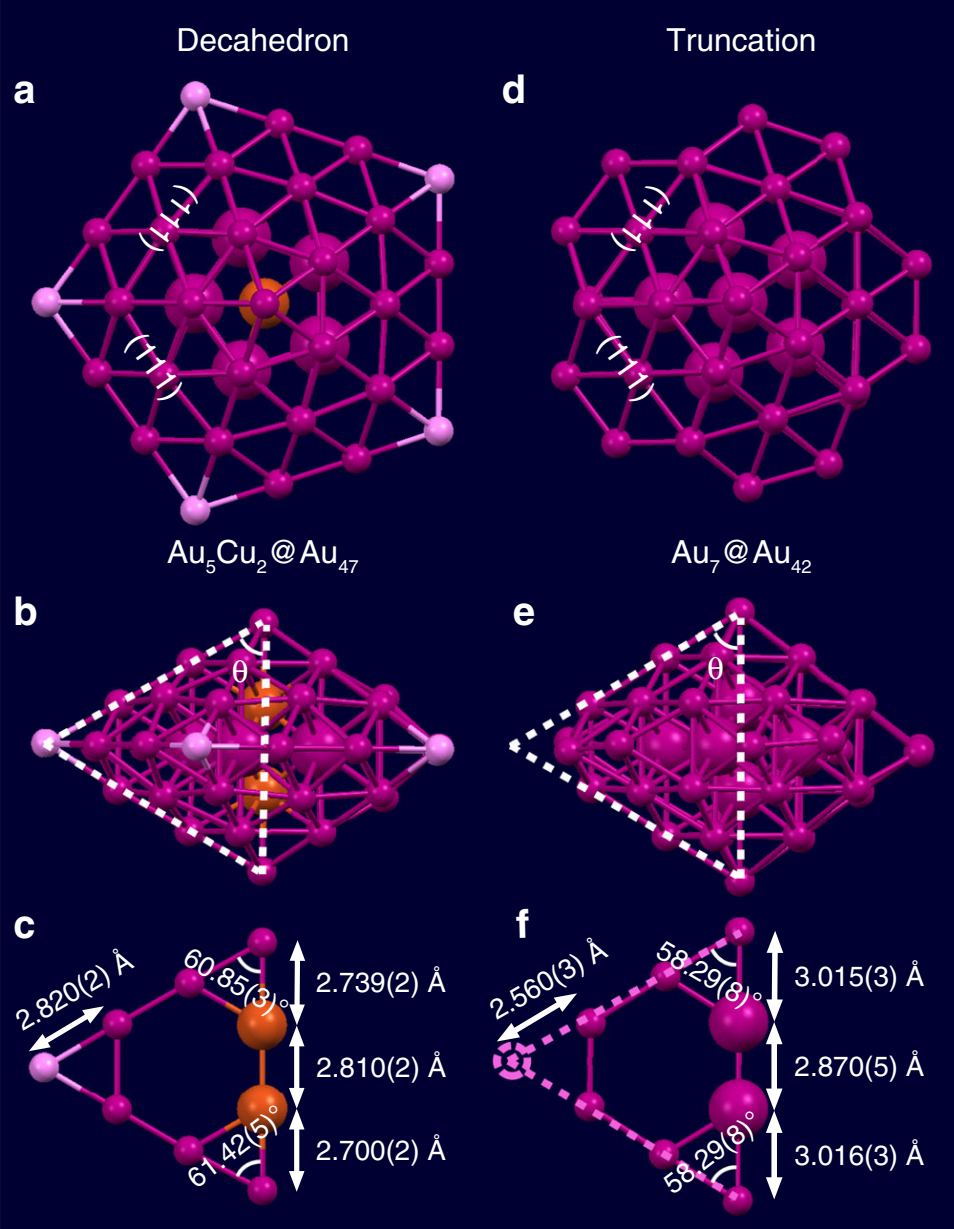
Comparison between the complete decahedron and the Marks decahedron. **a** Top view and **b** side view of the Au_5_Cu_2_@Au_47_ shells in [Cu_72_Au_52_(SR)_55_]^+^, and **c** the triangle formed by twinning edges and apex in the Au_47_ shell; **d** Top view and **e** side view of the Au_7_@Au_42_ shells in Au_102_(SR)_44_ or Au_103_S_2_(SR)_41_^16,17^, and **f** the truncated triangle formed by twinning edges and apex in the Au_42_ shell. Color labels: magenta/pink = Au, orange = Cu.

As shown in Fig. 2, we compared the two inner shells, i.e., Au_5_Cu_2_@Au_47_ (Fig. 2a, b) in [Cu_72_Au_52_(SR)_55_]^+^ and Au_7_@Au_42_ in Au_102_(SR)_44_ or Au_103_S_2_(SR)_41_ (Fig. 2d, e) to illustrate the difference brought by the copper incorporation. The parts marked by dashed triangles are emphasized in Fig. 2c, f, showing that the *C*_5_ axial Au–Cu–Cu–Au length (8.249(2) Å) is contracted by 7% compared to the Au–Au–Au–Au length (8.901(4) Å). In addition, the angle (θ) is also expanded for the Cu-doped kernel (see labels in Fig. 2c, f). Therefore, when extrapolating the two edges of the decahedron to reach a joint point, the Cu-doped structure gives a corner-to-neighbor distance of 2.820(2) Å, which matches with typical Au–Au bond length (2.88 Å) so that the five corner atoms can exist (Fig. 2c), but the homogold structure gives a much shorter corner-to-neighbor distance (2.560(3) Å), being much shorter than any realistic Au–Au bond length (Fig. 2f), thus the corner Au atoms have to be eliminated. This explains why the Au–Cu nanoalloy herein possesses a full decahedral kernel, while Au_102_ and Au_103_ structures have the Marks truncation. Taken together, the incorporation of two Cu atoms into the inner M_7_ kernel in the Cu_52_Au_72_ nanoalloy is critical for the formation of the full decahedral kernel. The two Cu atoms along the disclination core is also reasonable from a strain-induced segregation perspective in bimetallic multiply-twinned NPs in which a segregation of smaller atoms to the kernel could be noticeable^37^. It is worth mentioning that a similar Ag_7_@Ag_47_ kernel (Supplementary Fig. 10) was observed in Ag_136_^30^, comparing to the Au_5_Cu_2_@Au_47_ in Cu_72_Au_52_ herein. The comparison between Ag and Au NPs reinforces the effect that the two doping Cu atoms play a key role in reaching the full decahedral structure of the Au–Cu nanoalloy.

### Cu-thiolate cage resembling Penrose tiling

**Fig. 3:**
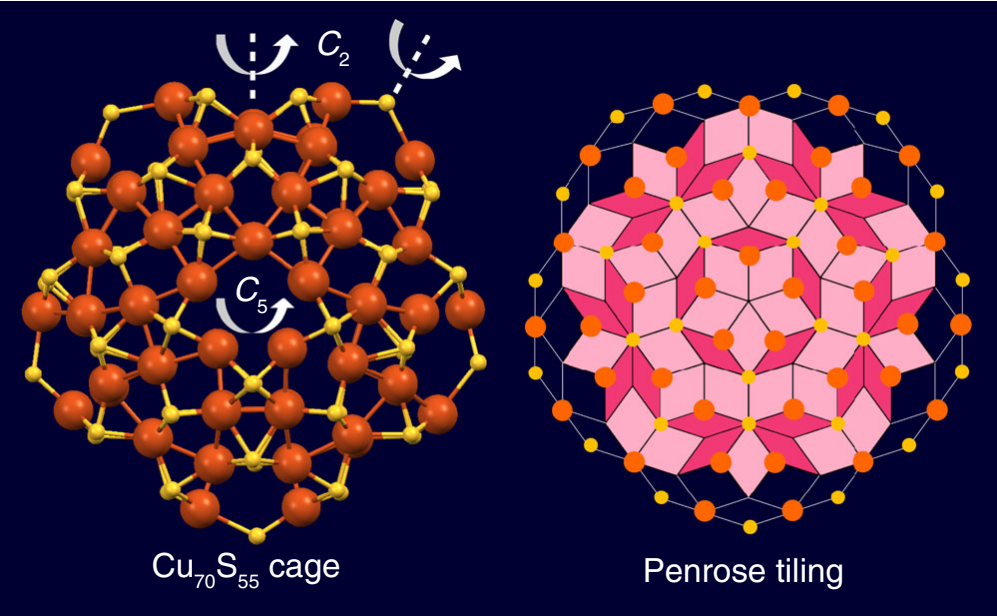
Structure of the Cu_70_S_55_ cage. Top view along the *C*_5_ axis (left); and a Penrose tiling with joint points marked by orange and yellow solid circles to represent Cu and S, respectively (right). Color labels: orange = Cu, yellow = S.

The formation of a full decahedron, Au_5_Cu_2_@Au_47_, further dictates the formation of the outermost Cu_70_(SR)_55_ cage with a flower-shaped surface pattern (Fig. 3). It is rationalized that the high symmetry (*D*_5h_) of the perfect Cu-thiolate cage is related to the highly coordinated sulfur atoms (*μ*_3_ or *μ*_4_), note: no lower coordinated thiolate (*μ*_2_) is involved in [Au_52_Cu_72_(SR)_55_]^+^ as in thiolate-protected homogold NPs^19^. This means that staple motifs or bridging thiolates—which are commonly observed in thiolate-protected gold NPs—are eliminated in the Cu-thiolate cage observed in the current work. It should be noted that the S atoms at the five tips of the flower petals are all coordinated with two Cu atoms and one Au atom inside. As a result, the cage is fixed well on the inner decahedron with much less relaxation, so as to maintain the horizontal mirror plane, but such a plane is missing in all gold cases^16–19,23^, thus, the [Au_52_Cu_72_(SR)_55_]^+^ nanoalloy remains achiral as the kernel is.

The highly regular structure of the Cu_70_S_55_ cage along the *C*_5_ axis reminds us of a Penrose tiling, an important mathematical and architectural model of aperiodic tessellations exhibiting both reflectional and rotational symmetry but without translational symmetry. The Penrose tiling (Fig. 3 right) has two sets of congruent prototiles, i.e., a fat rhombus (light pink) and a thin rhombus (dark pink). When we mark the joint points with solid circles representing Cu (orange) and S (yellow) atoms, respectively, they match very well with the Cu-thiolate cage except some distortions at the perimeter, probably because the Penrose tiling is a two-dimensional (2D) plane, while the cage on the particle is a 3D structure. The stereoscopic construction of perfection revealed by intrinsic packing pattern of atoms demonstrates how the aperiodic close-packing can evolve from two-dimension to three-dimension.

### Assembly of [Cu_72_Au_52_(SR)_55_]^+^Cl^−^ into single crystals

We further discuss the effect of the high *D*_5h_ symmetry on the particle assembly. In previous work, intriguing C–H···π interactions were observed between the ligands of neighboring particles, i.e., the interaction of C–H bonds from the phenyl rings or the methyl groups with the π electrons of the phenyl rings^19^. Specifically, side-by-side and point-to-point stackings of ligands were found in Au_246_(SR)_80_^19^, and the herringbone pattern of ligands led to a zigzag arrangement of particles in the crystal of Au_103_S_2_(SR)_41_^17^. Such patterns are essential to stabilize the macroscopic crystals because of much closer packing of particles into a regular pattern in the crystal. When the particle has an even higher symmetry than Au_246_(SR)_80_, we expect such interactions to be even stronger. Indeed, we found that in the assembly of [Cu_72_Au_52_(SR)_55_]^+^ particles into macroscopic crystals, the interparticle distances are as short as ~2.6 nm, much smaller than the diameter of the pentagon (~3.0 nm, Supplementary Fig. 11), which implies significant C–H···π interactions.

**Fig. 4:**
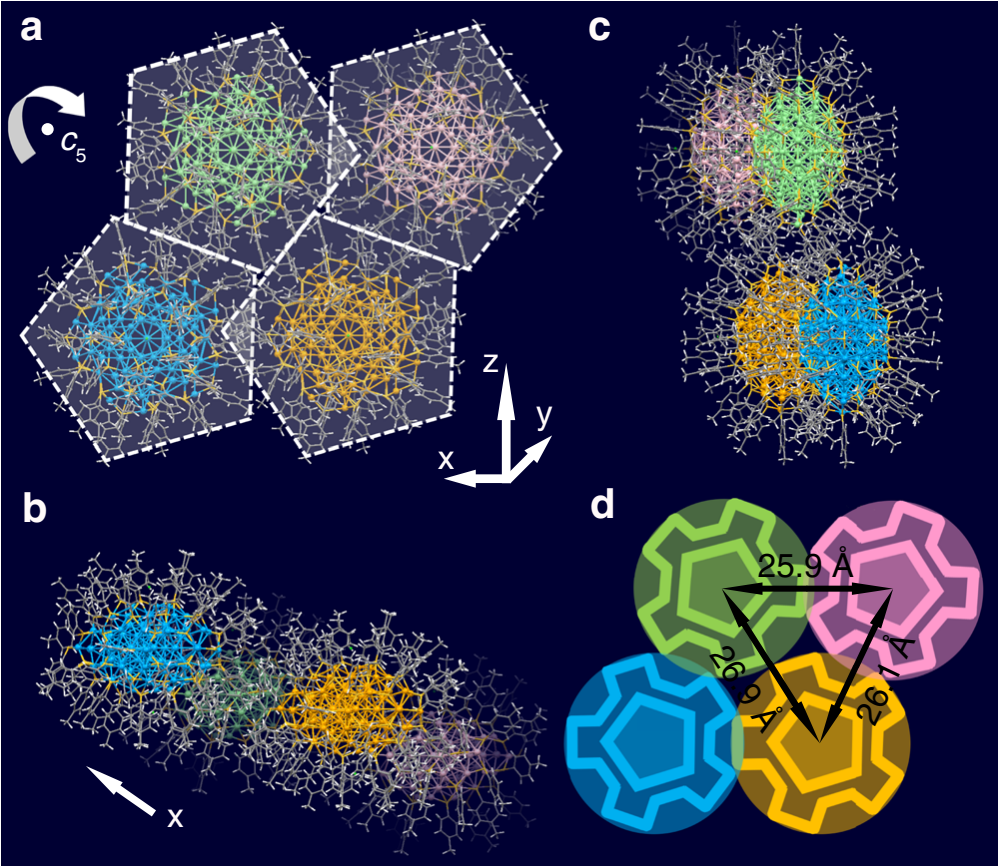
The assembly of [Cu_72_Au_52_(SR)_55_]^+^. **a** Four nearest pentagonal [Cu_72_Au_52_(SR)_55_]^+^ particles with tip-to-edge, edge-to-edge, and tip-to-tip interactions; **b**, **c** top and side views of the four interacting particles; **d** the “quadruple-gear” interlock diagram demonstrating the interactions among the four nearest particles.

As shown in Fig. 4a, the four nearest particles are marked by different colors. For the two particles arranged along the *x* direction, their ligands show a tip-to-edge interaction (green and pink particles, or light orange and blue particles); while for the two particles arranged along the z direction, their ligands show an edge-to-edge interaction (green and blue particles, or light orange and pink particles). The two particles at diagonal positions approach each other in a tip-to-tip manner (green and light orange particles). Figure 4b, c shows the four particles from different views. Such interweaving interactions remind us of the “quadruple-gear” meshing mechanism in which the rotation of any of the gears would lead to an integrated movement. However, the two particles at diagonal positions (green and light orange particles) also show C–H···π interactions between their ligands, making the four gears interlock with each other (Fig. 4d).

To be specific, the two approaching particles in an edge-to-edge assembly show significant intra- and interparticle interactions of the ligands (Supplementary Fig. 12). Interestingly, the intraparticle interactions (indicated by blue dashed lines) are all coming from methyl H to phenyl ring, while the interparticle interactions (indicated by yellow dashed lines) are all originated from phenyl ring H to phenyl ring. On a whole, the six ligands belong to two particles form a triangular mosaic pattern, which is even more compact than the herringbone pattern^17^. The interparticle interactions between phenyl ring H and phenyl ring for the two tip-to-tip approaching particles are shown in Supplementary Fig. 13. As for the two particles approaching each other in a tip-to-edge way, interparticle and intraparticle interactions can be observed as well, and consistently, the intraparticle interactions are still from phenyl ring H to phenyl ring interaction, while the interparticle interaction is due to the close methyl H to phenyl ring (Supplementary Fig. 14).

Moreover, this interlock pattern can repeat along the *x*-axis to a long range (Supplementary Fig. 15), but along *y*- or *z*-axis (Supplementary Figs. 14 and 15) the particles belonging to different sets have essentially no interaction. Thus, the single crystal can be considered as a bunching of nanowires along the *x*-axis, and each nanowire (marked in circles) is composed of two lines (x and x’, Supplementary Figs. 16 and 17) of highly connected particles, resembling fibrous construction, which might endow the single crystal with interesting anisotropic electrical transport properties^38^. It is expected that such twin nanowires with larger cross-sections would increase the conductivity of the material for potential applications.

## Discussion

The Au_52_Cu_72_(*p*-MBT)_55_ nanoalloy offers several important features and implications. The first feature is the unusual behavior that two copper atoms occupy the central positions of this nanoalloy. Such a localization is interesting and differs from the random Au_1-*x*_Cu_*x*_ alloys as well as the ordered phases of Au_3_Cu_1_, Au_1_Cu_1_, and Au_1_Cu_3_ in bulk and nanoparticles^39,40^. Localization of the two Cu atoms is critical to realize a perfect decahedral kernel without truncation, i.e., the innermost Au_5_Cu_2_ dictating a subsequent decahedral Au_47_ shell, in contrast with the Au_7_ kernel dictating a truncated Au_42_ shell in homogold systems. A scrutiny of local bond distances and a comparison with Marks-decahedra in homogold systems reveal that the two copper atoms shorten the overall length of the central axis so as to give enough space for the five Au atoms at the corners of the pentagon, but this is not the case in homogold NPs. Such atomic-level insights for the truncation of Marks decahedron are unprecedented, which could not be obtained by high-resolution electron microscopy analysis^41,42^. Future work may explore the Au:Cu ratio to tailor the composition and investigate the potential Cu-atom number dependent structural reorganization predicted by theoretical simulations^42^.

The Au_52_Cu_72_ nanoalloy also exhibits the highest symmetry (*D*_5h_) among the reported ones so far. The particle shape is indeed not predicted for Au–Cu alloy nanoparticles, as the most stable shapes were predicted to be dodecahedron, truncated octahedron, and icosahedron regardless of the composition and the size of the nanoalloy^28^. With respect to the surface protection of Au_52_Cu_72_, the discovered Cu surface is also opposite to the earlier prediction of Cu(core)-Au(surface) for all sizes and shapes of nanoparticles^43^. Previous calculations showed that the lower surface energy of Au should render Au segregation to the surface in AuCu alloys while copper segregates at subsurface sites^44^. We rationalize that the opposite trend observed in our work should be due to the Cu-S bonding, because the previous prediction considered only the fact that the larger Au atom than Cu requires fewer atoms in covering the particle surface, hence, fewer broken bonds and lower surface energy^43^. The outmost shell—an extended, rigid Cu_70_(SR)_55_ cage—resembles an aesthetic 3D Penrose tiling, which is the key to retain the high symmetry. Compared to the commonly observed discrete staple motifs or bridging thiolates made up of low-coordinated *μ*_2_-S in Au-thiolate systems, the Cu-thiolate cage is composed of all highly coordinated sulfur (*μ*_3_ or *μ*_4_), with very little relaxation, and thus, making this nanoalloy achiral.

Intraparticle and interparticle interactions of ligands are found to be significant in the assembly of [Au_52_Cu_72_(*p*-MBT)_55_] due to its unique symmetry, and a “quadruple-gear” interlocking pattern is observed among four nearest particles. Such mechanistic insights provide implications on how to tailor the particle symmetry in order to control the macroscopic assembly, and the obtained single crystals may find important applications involving anisotropic electron transport properties.

Alloying of copper with gold also largely improves the stability, which has been widely adopted in nanoscience research^43^, but atomic-level information of Cu distributions (i.e., kernel vs surface) could not be obtained in earlier work. The atomically resolved Au_52_Cu_72_ nanoalloy may provide a model for rationalization of the structure, stability, synergism, and catalytic properties of Au–Cu alloy nanoparticles^28,45^.

Overall, the Au_52_Cu_72_ nanoalloy demonstrates on how to manipulate the kernel shape of Au by heterometal incorporation and emphasizes the relationship between the particle symmetry and particle assembly at the atomic level, which is expected to open up new opportunities in future research on Au–Cu alloys and their applications in catalysis, photonics, antibacterial, and biomedicine fields^38–46^.

## Methods

### Reagents

All reagents and solvents were commercially available and used as received without further purification, including *para*-methylphenylthiophenol (C_7_H_7_SH, ≥99.9%, Aladdin), 4-*tert*-butylbenzenethiol (TBBT, 97%, Alfa Aesar), tetrachloroauric(III) acid (HAuCl_4_·3H_2_O, ≥99.99% metals basis, Energy Chemical), tetraoctylammonium bromide (TOAB, ≥98%, Aladdin), sodium borohydride (NaBH_4_, ≥98%, Energy Chemical), dichloromethane (CH_2_Cl_2_, ≥98%, Aladdin), toluene (C_6_H_6_, HPLC, Aladdin), methanol (CH_3_OH, ≥98%, Aladdin), ethanol (CH_3_CH_2_OH, ≥98%, Aladdin), triphenylphosphine (PPh_3_, ≥98.8%, Energy Chemical), copper chloride (II) (CuCl_2_, ≥98.8%, Alfa Aesar), acetonitrile (CH_3_CN, ≥98%, Aladdin), and pure water.

### Synthesis of [Au_52_Cu_72_(*p*-MBT)_55_]^+^ nanoalloys

Briefly, 0.10 g HAuCl_4_·3H_2_O was dissolved in 5 mL nanopure water, and 0.16 g TOAB was dissolved in 30 mL toluene. The two solutions were combined in a 100 mL tri-neck round bottom flask. The solution was vigorously stirred (~1100 rpm) with a magnetic stir bar to facilitate phase transfer of Au(III) salt into the organic phase. After ~30 min, phase transfer was completed, leaving a clear aqueous phase at the bottom of the flask, which was then removed. After that, 0.10 g PPh_3_ was added into the dichloromethane solution of Au(III), and the color of the solution changed from orange to colorless. Then, 0.25 g CuCl_2_ was added to a dichloromethane solution. Subsequently, 15 mL CH_3_H_2_OH was added into the solution. After ~30 min, 0.35 mL *p*-MBT was added to the solution. After ~1 h, 5 mL aqueous solution of NaBH_4_ (150 mg) was rapidly added. The reaction was allowed to proceed for ~16 h. The product was washed several times with CH_3_CN to remove the redundant thiol, PPh_3_ and by-products until the optical absorption spectrum showed distinct peaks, which gave rise to pure [Cu_72_Au_52_(*p*-MBT)_55_]Cl nanoalloys. It is worth noting that the [Au_52_Cu_72_(TBBT)_55_]^+^ nanoalloy was obtained with the same method simply by changing the ligand from *p*-MBT to TBBT. The [Cu_72_Au_52_(*p*-MBT)_55_]SbF_6_ nanoalloy with different counterion (SbF_6_^−^) was also obtained by adding a methanol solution of NaSbF_6_ (5 mg) to a CH_2_Cl_2_ solution of [Cu_72_Au_52_(*p*-MBT)_55_]Cl (10 mg), followed by thorough mixing and then washing several times with CH_3_CN/H_2_O (v: v = 2: 1) to remove the redundant Na^+^, Cl^−^, and excess SbF_6_^−^ ions.

### Characterization

UV-vis spectral measurements were carried out with an Agilent HP8453 diode array spectrometer. The crystals of Au_52_Cu_72_ nanoalloys were re-dissolved in dichloromethane (CH_2_Cl_2_) for spectral measurements. TGA was carried out on a thermogravimetric analyzer (DTG-60H, Shimadzu Instruments, Inc.) with 4.613 mg of particles in a SiO_2_ pan at a heating rate of 10 °C min^−1^ in an Ar atmosphere. Two or three crystals of Au_52_Cu_72_ were picked and dissolved 0.5 mL fresh aqua regia. After ultrasonic dissolution for 3 h, the 5 ml ultrapure water was added into above solution, which can be used to test with ICP-MS. Electrospray ionization (ESI) mass spectra were acquired using a Bruker Q-TOF mass spectrometer equipped with ESI source. The sample was dissolved in methylbenzene (~1 mg ∙ ml^−1^) and then mixed with a dry methanol solution of CsOAc (30 mM) by a 3:1 vol ratio. The sample was infused at 180 μL ∙ h^−1^ directly. The source temperature was kept at 50 °C with the spray voltage keeping at 4 kV.

### X-ray crystallographic determination

Black, rod-like crystals were obtained by diffusing CH_3_CN into a toluene solution of NPs for 6–10 days at 10 °C. A suitable crystal was selected and performed on a Bruker D8 Venture with GaKα radiation (λ = 1.34139 Å). The crystal was kept at 123 K during data collection. Using Olex2^47^, the structure was solved with the olex2.solve^48^ structure solution program using Charge Flipping and refined with the olex2.refine^49^ refinement package using Gauss-Newton minimisation. All the Au, Cu, Cl, and S atoms were found directly. Remaining nonhydrogen atoms were generated via subsequent difference Fourier syntheses. All the nonhydrogen atoms were refined anisotropically. The center was resided by two Cu atoms, and their ellipsoids largely extend and are abnormal when partially occupied by Au atoms. The peripheral atoms possess the same conditions. All the hydrogen atoms were set in geometrically calculated positions and refined isotropically using a riding model. The diffuse electron densities resulting from the residual solvent molecules were removed from the dataset using the SQUEEZE routine of PLATON and refined further using the data generated.

### Crystal data

for C_385_H_385_Au_52_ClCu_72_S_55_ (*M* *=* 21627.80 g mol^−1^): triclinic, space group P-1 (no. 2), *a* = 25.8880(19) Å, *b* = 26.7997(18) Å, *c* = 45.013(3) Å, *α* = 79.828(2)°, *β* = 85.255(3)°, *γ* = 69.166(2), *V* *=* 28722(3) Å3, *Z* = 2, *T* = 123(2) K, μ(GaKα) = 32.088 mm^−1^, *D*_*calc*_ = 2.501 g cm^−3^, 356930 reflections measured (1.736° ≤ 2*θ* ≤ 110.384°), 108535 unique (*R*_int_ = 0.0445, *R*_sigma_ = 0.0475), which were used in all calculations. The final *R*_1_ was 0.0388 (I > 2σ(I)) and *wR*_2_ was 0.1206 (all data).

## Supplementary information

Supplementary Information

## Data Availability

The X-ray crystallographic coordinates for structures reported in this study (see Supplementary Table 1 and Supplementary Data 1) have been deposited at the Cambridge Crystallographic Data Centre (CCDC), under deposition number CCDC 1875255. These data can be obtained free of charge from The Cambridge Crystallographic Data Centre via www.ccdc.cam.ac.uk/data_request/cif.
