# Supplementary material for: Atomically resolved Au52Cu72(SR)55 nanoalloy reveals Marks decahedron truncation and Penrose tiling surface
Source: Nat Commun. 2020 Jan 24;11:478. doi: 10.1038/s41467-020-14400-2 (PMC6981204; doi:10.1038/s41467-020-14400-2)
Supplement: Supplementary file 1 — Supplementary Information [file 41467_2020_14400_MOESM1_ESM.pdf]

**Atomically resolved $Au_{52}Cu_{72}(SR)_{55}$ nanoalloy reveals Marks decahedron truncation and Penrose tiling surface**

Song *et al*

**Supplementary Table 1** Crystal data and structure refinement for $[Au_{52}Cu_{72}(p\text{-MBT})_{55}]^+Cl^-$.

| Identification code | $Cu_{72}Au_{52}$ |
|---|---|
| Empirical formula | $C_{385}H_{385}Au_{52}ClCu_{72}S_{55}$ |
| Formula weight | 21627.80 |
| Temperature/K | 123 |
| Crystal system | triclinic |
| Space group | $P\bar{1}$ |
| a/Å | 25.8880(19) |
| b/Å | 26.7997(18) |
| c/Å | 45.013(3) |
| α/° | 79.828(2) |
| β/° | 85.255(3) |
| γ/° | 69.166(2) |
| Volume/Å$^3$ | 28722(3) |
| Z | 2 |
| $\rho_{calc}$g/cm$^3$ | 2.501 |
| μ/mm$^{-1}$ | 32.176 |
| F(000) | 19576.0 |
| Crystal size/mm$^3$ | 0.42 × 0.22 × 0.20 |
| Radiation | $GaK_{\alpha}$ (λ = 1.34139) |
| 2Θ range for data collection/° | 1.736 to 110.384 |
| Index ranges | $-31 \leq h \leq 31$, $-32 \leq k \leq 32$, $-40 \leq l \leq 54$ |
| Reflections collected | 356930 |
| Independent reflections | 108535 [$R_{int}$ = 0.0445, $R_{sigma}$ = 0.0475] |
| Data/restraints/parameters | 108535/0/5042 |
| Goodness-of-fit on $F^2$ | 1.090 |
| Final R indexes [I>=2σ (I)] | $R_1$ = 0.0388, $wR_2$ = 0.1090 |
| Final R indexes [all data] | $R_1$ = 0.0525, $wR_2$ = 0.1146 |
| Largest diff. peak/hole / e Å$^{-3}$ | 3.92/-2.15 |

**Supplementary Table 2** The result of ICP-MS analysis on the $[Cu_{72}Au_{52}(p\text{-MBT})_{55}]^+$ nanoalloy.

| | Cu (ppm) | Au (ppm) | Cu : Au (mol) |
|---|---|---|---|
| **$Cu_{72}Au_{52}$** | 91.38 | 204.34 | 72 : 51.89 |
| | 40.43 | 90.46 | 72 : 51.92 |
| | 105.69 | 237.21 | 72 : 52.08 |

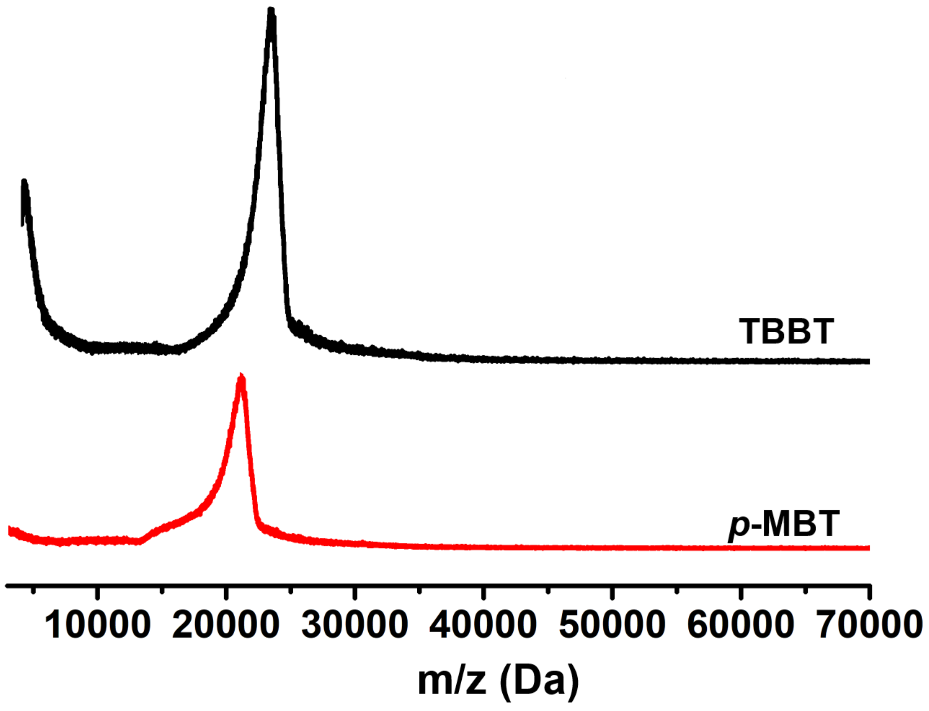


**Supplementary Fig 1** MALDI mass spectra of $[Au_{52}Cu_{72}(SR)_{55}]^+$ capped by *p*-MBT (red) or TBBT (black) in positive mode (TBBT = SPh-$^t$Bu and *p*-MBT = SPh-*p*-$CH_3$).

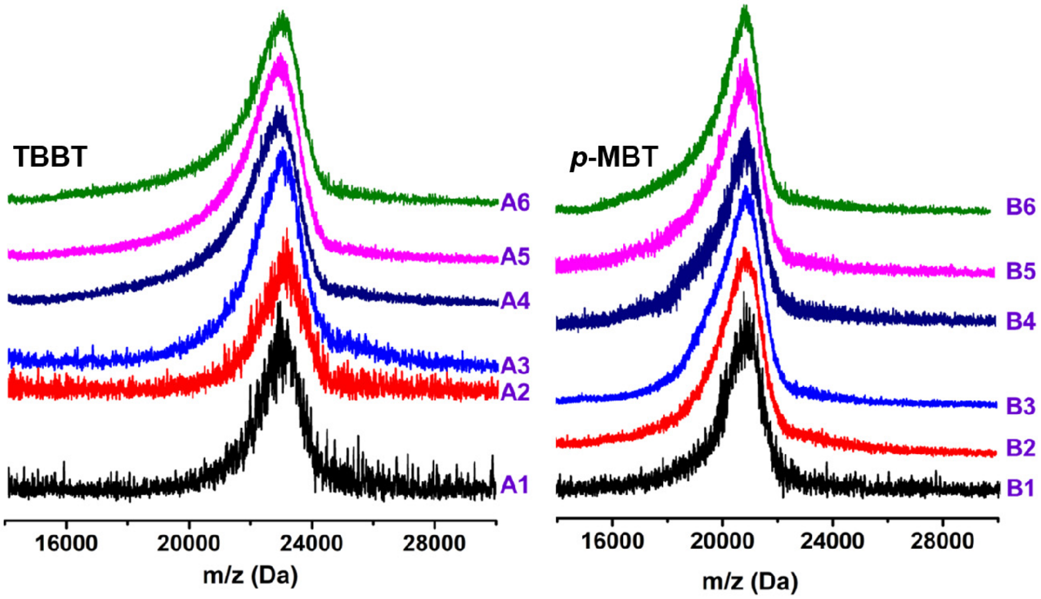


**Supplementary Fig 2** Intensity-dependent MALDI mass spectra of $[Au_{52}Cu_{72}(TBBT)_{55}]^+$ (left panel) and $[Au_{52}Cu_{72}(p\text{-MBT})_{55}]^+$ (right panel) nanoalloys. The different parameters (A1/B1-A5/B5) in MALDI-MS are listed in Table S3.

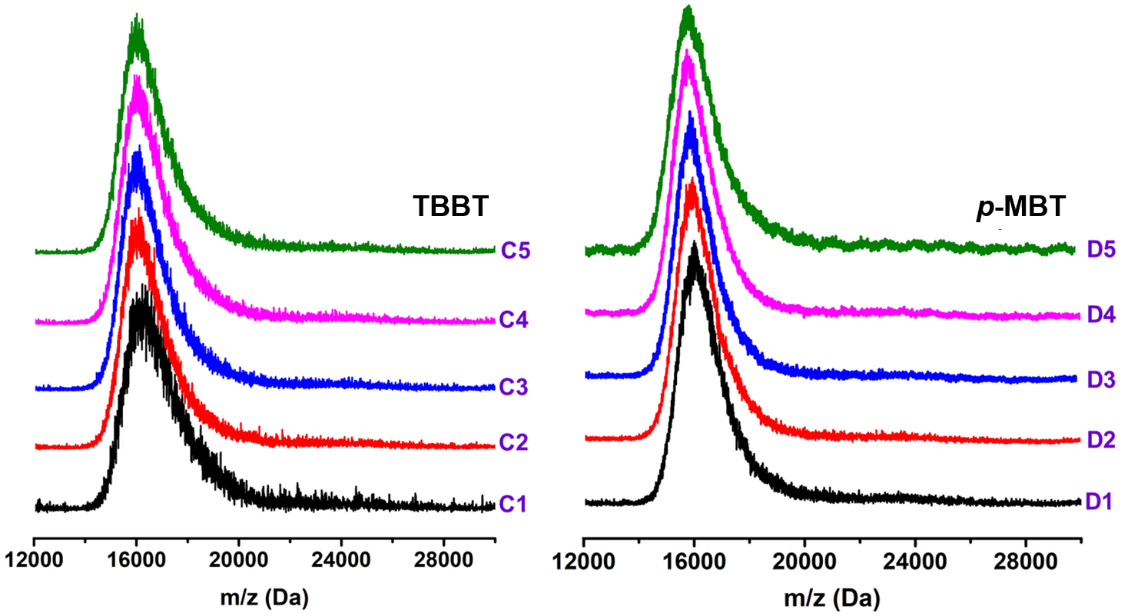


**Supplementary Fig 3** Intensity-dependent LDI mass spectra of $[Au_{52}Cu_{72}(TBBT)_{55}]^+$ (left) and $[Au_{52}Cu_{72}(p\text{-MBT})_{55}]^+$ (right) nanoalloys. The different parameters (C1/D1-C5/D5) in LDI-MS are listed in Table S3.

**Supplementary Table 3** Different parameters in MALDI/LDI-MS

| | A1/B1 | A2/B2 | A3/B3 | A4/B4 | A5/B5 | A6/B6 | C1/D1 | C2/D2 | C3/D3 | C4/D4 | C5/D5 |
|---|---|---|---|---|---|---|---|---|---|---|---|
| Offset | 55% | 60% | 70% | 80% | 90% | 70% | 60% | 70% | 80% | 90% | 70% |
| Range | 20% | 20% | 20% | 20% | 10% | 30% | 20% | 20% | 20% | 10% | 30% |
| Linear | 90X | 90X | 90X | 90X | 90X | 90X | 90X | 90X | 90X | 90X | 90X |
| Percent | 100% | 100% | 100% | 100% | 100% | 100% | 100% | 100% | 100% | 100% | 100% |

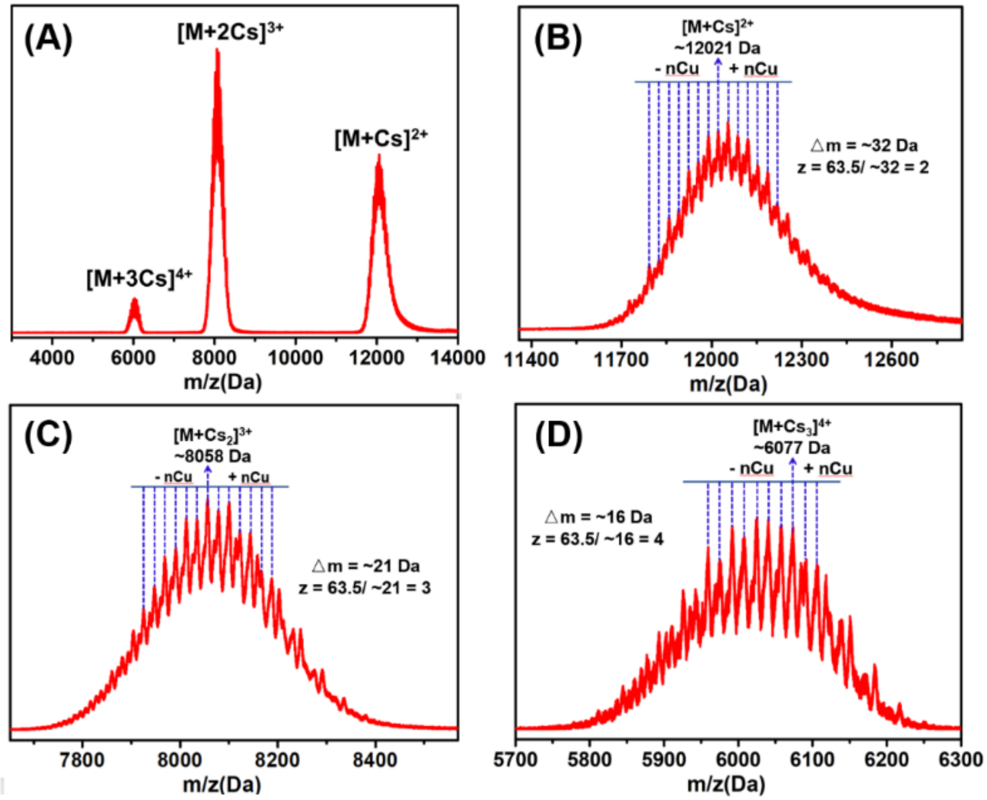


**Supplementary Fig 4** The ESI mass spectrum of the $[Au_{52}Cu_{72}(TBBT)_{55}]^+$ in positive mode by adding $Cs^+$. (A) Full spectrum, (B) Zoom-in of the 2+ ion set, (C) Zoom-in of the 3+ ion set, and (D) Zoom-in of the 4+ ion set.

**Supplementary Table 4** The assignments of the peaks in each set in the ESI mass spectrum.

| **Peaks in set a** | | **Peaks in set b** | | **Peaks in set c** | |
|---|---|---|---|---|---|
| value | Assigned formula | value | Assigned formula | value | Assigned formula |
| 12178 | $[Au_{52}Cu_{77}(SR)_{55}Cs]^{2+}$ | 8163 | $[Au_{52}Cu_{77}(SR)_{55}Cs_2]^{3+}$ | 6156 | $[Au_{52}Cu_{77}(SR)_{55}Cs_3]^{4+}$ |
| 12147 | $[Au_{52}Cu_{76}(SR)_{55}Cs]^{2+}$ | 8142 | $[Au_{52}Cu_{76}(SR)_{55}Cs_2]^{3+}$ | 6140 | $[Au_{52}Cu_{76}(SR)_{55}Cs_3]^{4+}$ |
| 12115 | $[Au_{52}Cu_{75}(SR)_{55}Cs]^{2+}$ | 8121 | $[Au_{52}Cu_{75}(SR)_{55}Cs_2]^{3+}$ | 6124 | $[Au_{52}Cu_{75}(SR)_{55}Cs_3]^{4+}$ |
| 12083 | $[Au_{52}Cu_{74}(SR)_{55}Cs]^{2+}$ | 8099 | $[Au_{52}Cu_{74}(SR)_{55}Cs_2]^{3+}$ | 6108 | $[Au_{52}Cu_{74}(SR)_{55}Cs_3]^{4+}$ |
| 12052 | $[Au_{52}Cu_{73}(SR)_{55}Cs]^{2+}$ | 8078 | $[Au_{52}Cu_{73}(SR)_{55}Cs_2]^{3+}$ | 6092 | $[Au_{52}Cu_{73}(SR)_{55}Cs_3]^{4+}$ |
| **12021** | $\mathbf{[Au_{52}Cu_{72}(SR)_{55}Cs]^{2+}}$ | **8058** | $\mathbf{[Au_{52}Cu_{72}(SR)_{55}Cs_2]^{3+}}$ | **6077** | $\mathbf{[Au_{52}Cu_{72}(SR)_{55}Cs_3]^{4+}}$ |
| 11988 | $[Au_{52}Cu_{71}(SR)_{55}Cs]^{2++}$ | 8036 | $[Au_{52}Cu_{71}(SR)_{55}Cs_2]^{3+}$ | 6060 | $[Au_{52}Cu_{71}(SR)_{55}Cs_3]^{4+}$ |
| 11956 | $[Au_{52}Cu_{70}(SR)_{55}Cs]^{2+}$ | 8015 | $[Au_{52}Cu_{70}(SR)_{55}Cs_2]^{3+}$ | 6044 | $[Au_{52}Cu_{70}(SR)_{55}Cs_3]^{4+}$ |
| 11924 | $[Au_{52}Cu_{69}(SR)_{55}Cs]^{2+}$ | 7994 | $[Au_{52}Cu_{69}(SR)_{55}Cs_2]^{3+}$ | 6029 | $[Au_{52}Cu_{69}(SR)_{55}Cs_3]^{4+}$ |
| 11893 | $[Au_{52}Cu_{68}(SR)_{55}Cs]^{2+}$ | 7973 | $[Au_{52}Cu_{68}(SR)_{55}Cs_2]^{3+}$ | 6013 | $[Au_{52}Cu_{68}(SR)_{55}Cs_3]^{4+}$ |
| 11861 | $[Au_{52}Cu_{67}(SR)_{55}Cs]^{2+}$ | 7951 | $[Au_{52}Cu_{67}(SR)_{55}Cs_2]^{3+}$ | 5997 | $[Au_{52}Cu_{67}(SR)_{55}Cs_3]^{4+}$ |

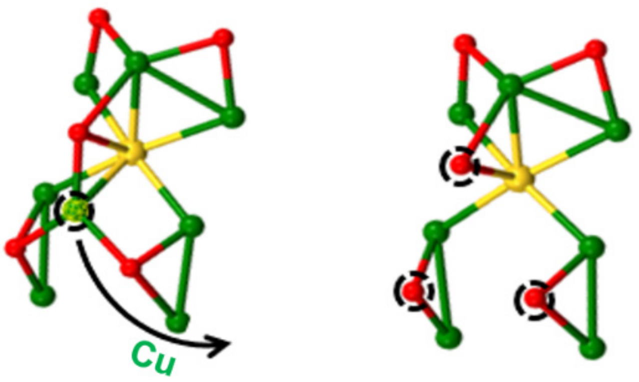


**Supplementary Fig 5** A possible pathway for losing surface Cu atoms under ESI-MS conditions (Cu: green, S: red).

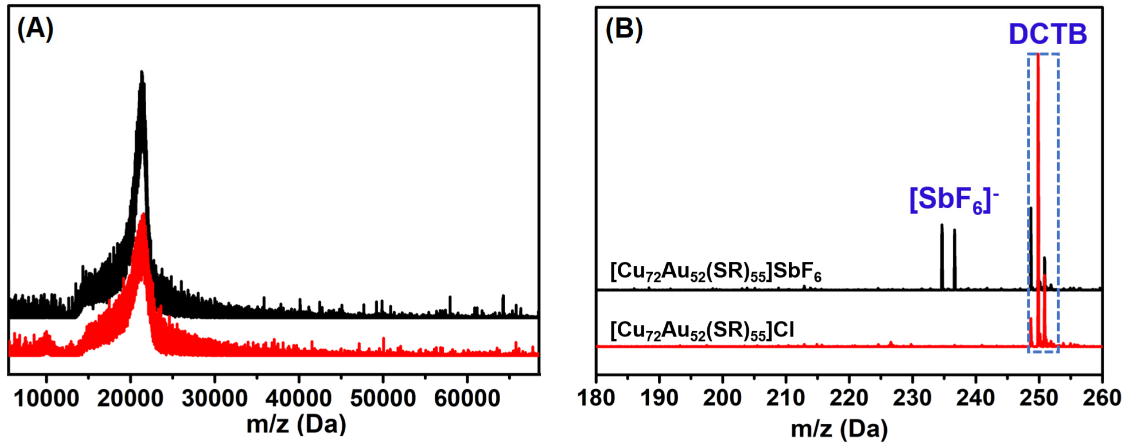


**Supplementary Fig 6** Experimental proof for the existence of $Cl^-$ in the $[Cu_{72}Au_{52}(p\text{-MBT})_{55}]Cl$ nanoalloys. (A) High-mass range spectra of $[Au_{52}Cu_{72}(p\text{-MBT})_{55}]Cl$ (red spectrum) and $[Au_{52}Cu_{72}(p\text{-MBT})_{55}]SbF_6$ (black); (B) low-mass range spectra, in which the peaks for $[SbF_6]^-$ were found in addition to those from the matrix (i.e., DCTB (*trans*-2-[3-(4-*tert*-Butylphenyl)-2-methyl-2-propenylidene]malononitrile)).

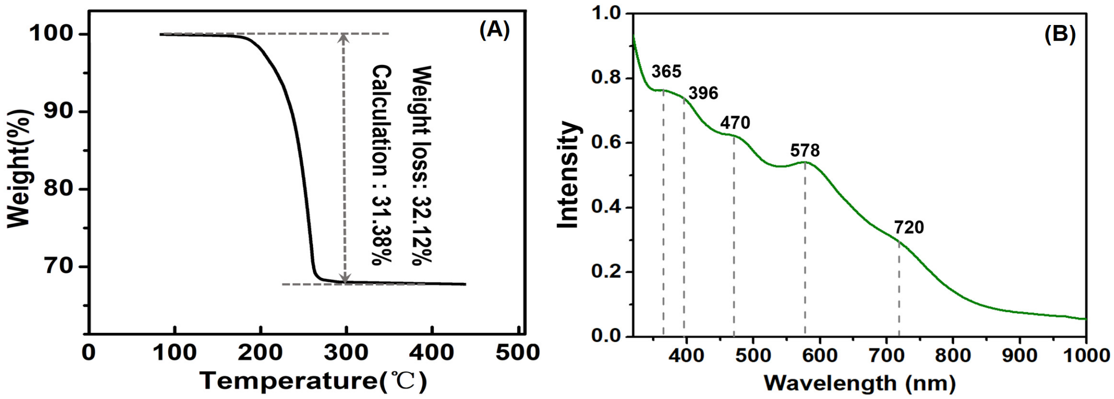


**Supplementary Fig 7** (A) The thermogravimetric analysis, and (B) The UV-vis absorption spectrum of $[Cu_{72}Au_{52}(p\text{-MBT})_{55}]^+$ nanoalloys.

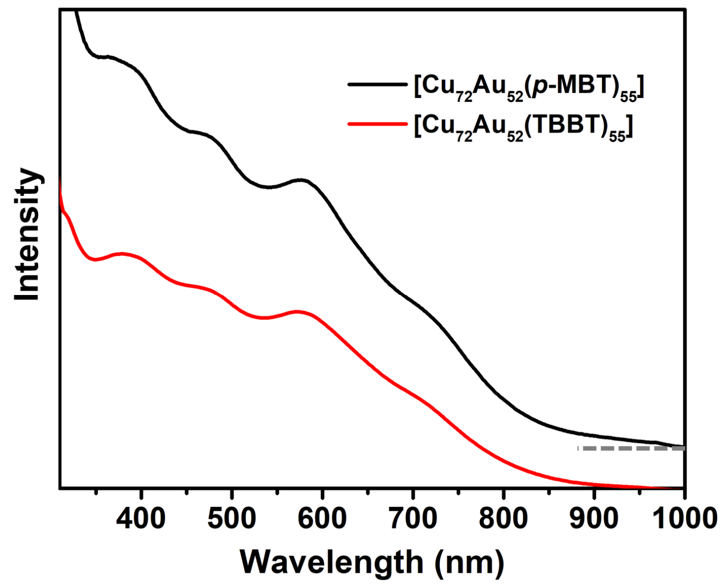


**Supplementary Fig 8** UV-vis absorption spectra of two $[Cu_{72}Au_{52}(SR)_{55}]^{+}$ nanoalloys protecting by different ligands.

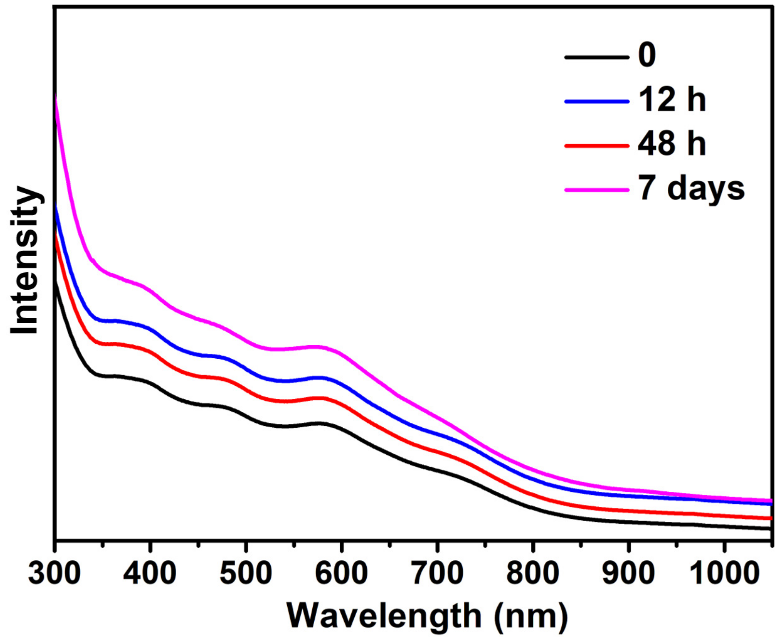


**Supplementary Fig 9** Time-dependent UV-vis absorption spectra of $[Cu_{72}Au_{52}(SR)_{55}]^{+}$ nanoalloys in $CH_2Cl_2$ in the air atmosphere (room temperature).

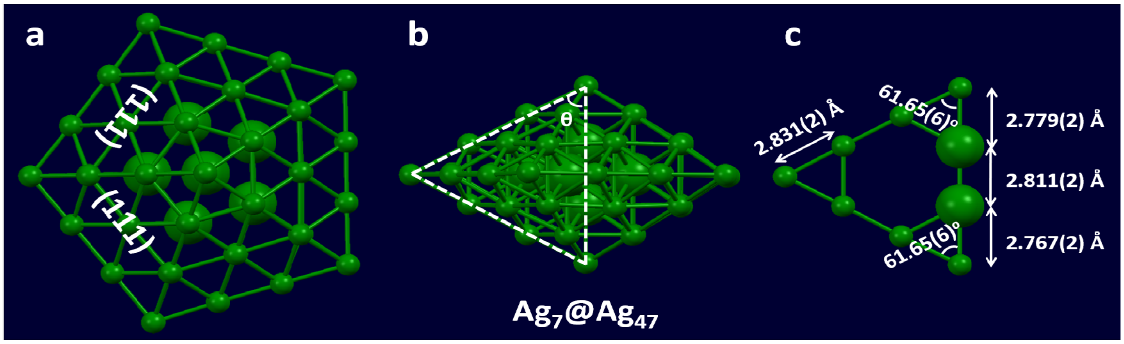


**Supplementary Fig 10** (a) top view and (b) side view of the $Ag_7@Ag_{47}$ shells in $Ag_{136}$ (ref. 30) and (c) the triangle formed by twinning edges and apex in the $Ag_{47}$ shell.

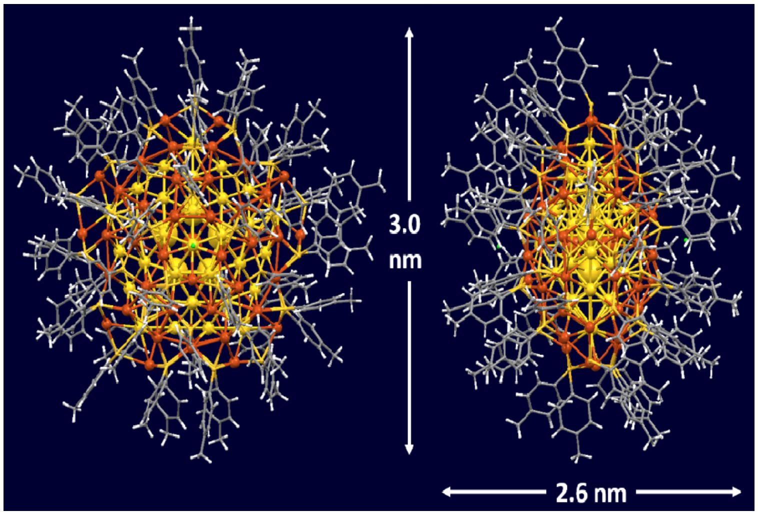


**Supplementary Fig 11** The overall size of $[Cu_{72}Au_{52}(SR)_{55}]^{+}$.

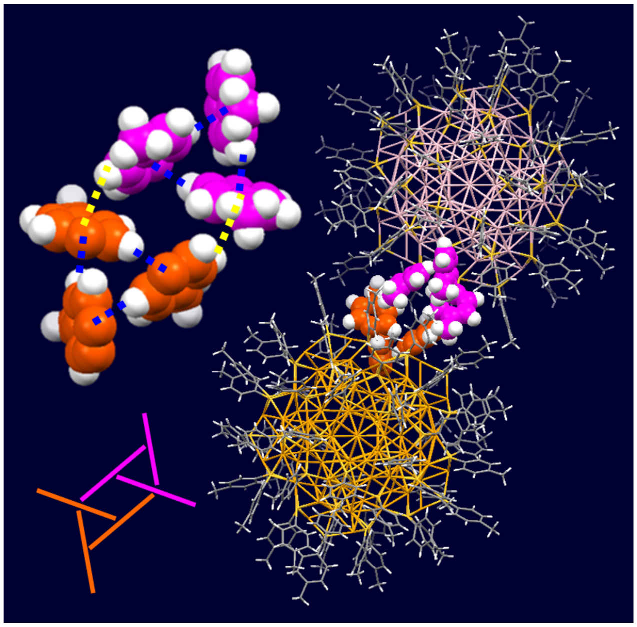

**Supplementary Fig 12** Intra- and inter-cluster interactions between two $[Cu_{72}Au_{52}(SR)_{55}]^{+}$ nanoalloy in an edge-to-edge approach, showing six interacting *p*-MBT ligands in a triangular mosaic pattern. Color labels: pink, light orange = Au/Cu, yellow = S, grey, magenta, orange = C, and white = H.

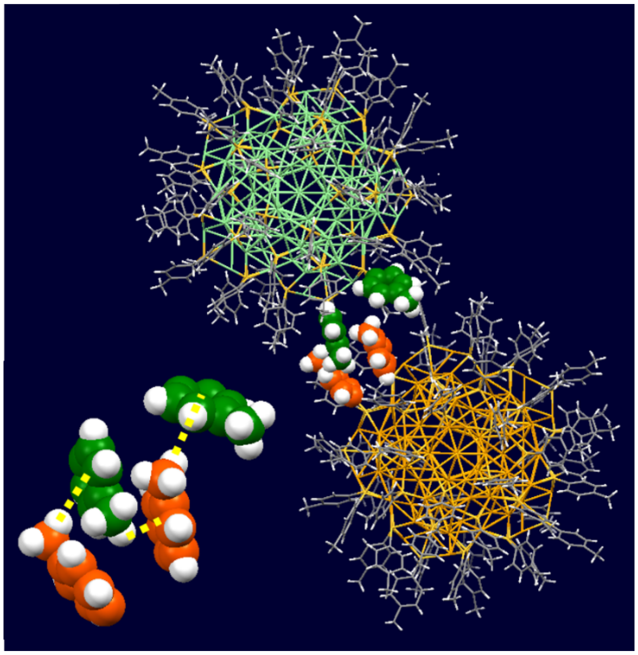

**Supplementary Fig 13** Inter-cluster interactions between two $[Cu_{72}Au_{52}(SR)_{55}]^{+}$ nanoalloys in a tip-to-tip approach. Color labels: light green, light orange = Au/Cu, yellow = S, grey, green, orange = C, and white = H.

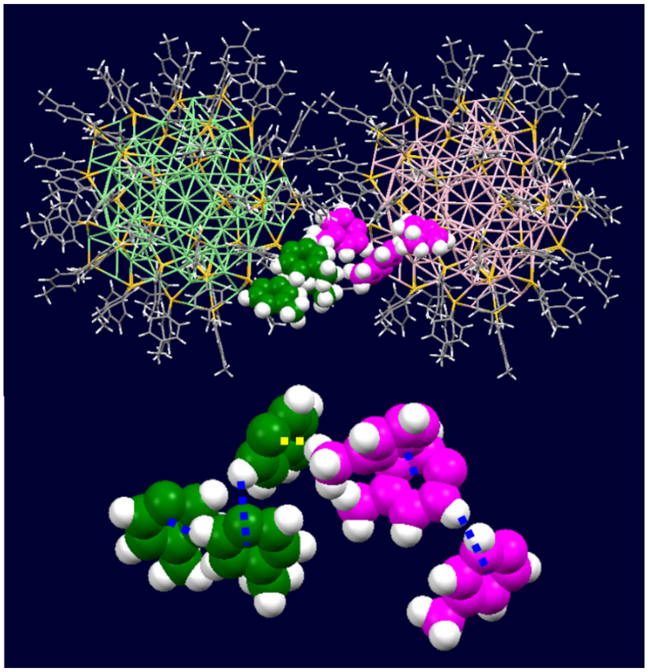

**Supplementary Fig 14** Intra- and inter-cluster interactions between two $[Cu_{72}Au_{52}(SR)_{55}]^+$ nanoalloy in a tip-to-edge approach. Color labels: light green, pink = Au/Cu, yellow = S, grey, green, magenta = C, and white = H.

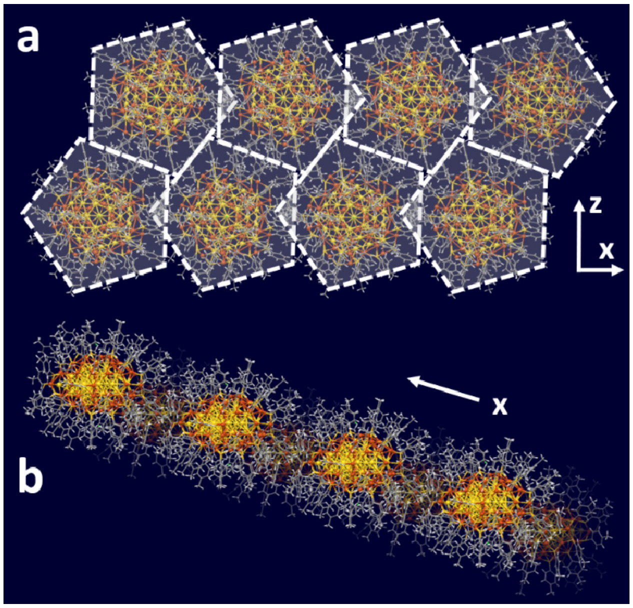


**Supplementary Fig 15** (a) front and (b) top view of the twin nanowire formed by interlocked $[Cu_{72}Au_{52}(SR)_{55}]^+$ nanoalloys along x axis.

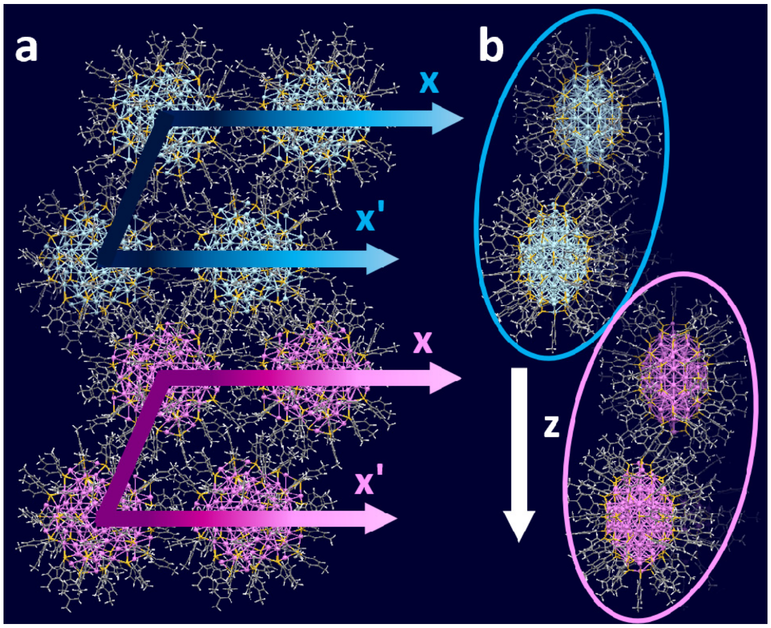


**Supplementary Fig 16** (a) front and (b) side view of the two sets of nanowires which are not interact along z axis. Each nanowire is composed of two lines (x and x') of interlocked $[Cu_{72}Au_{52}(SR)_{55}]^{+}$ nanoalloys.

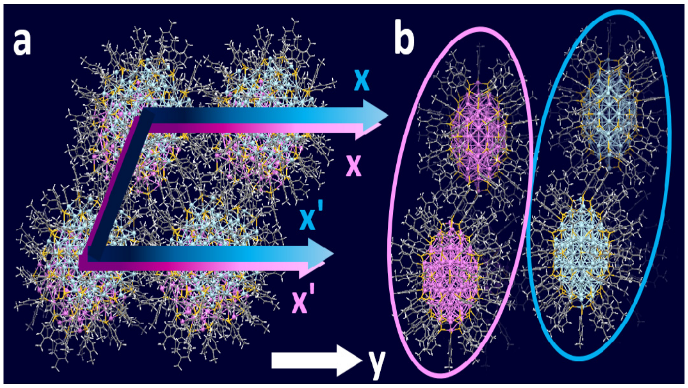


**Supplementary Fig 17** (a) front and (b) top view of the two sets of nanowires which are not interact along y axis. Each nanowire is composed of two lines (x and x') of interlocked $[Cu_{72}Au_{52}(SR)_{55}]^{+}$ nanoalloys.
